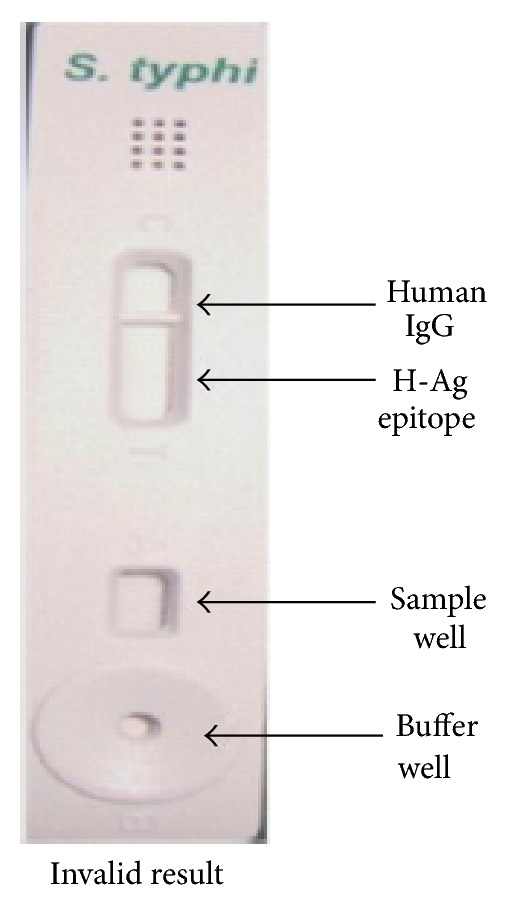# Development of a Novel Rapid Immunodiagnostic Kit Based on Flagellar 40 kDa Antigen Epitope for the Detection of Typhoid Fever in Indian Patients

**DOI:** 10.1155/2013/363652

**Published:** 2013-02-05

**Authors:** Rahul Mitra, Surya Bhan, Gopal Nath, Narender Kumar, Ziledar Ali

**Affiliations:** ^1^Department of Biochemistry, Institute of Medical Sciences, Banaras Hindu University, Varanasi 221005, India; ^2^Department of Biochemistry, NEHU, Shillong 793022, India; ^3^Department of Microbiology, Institute of Medical Sciences, Banaras Hindu University, Varanasi 221005, India; ^4^Department of Biochemistry, SRMS Institute of Medical Sciences, Bareilly 243202, India

## Abstract

To aid the clinical diagnosis of typhoid fever in India, where most hospitals and primary health centres have no facilities for culture, we report on the development of a novel and rapid immunodiagnostic kit for the direct detection of *Salmonella* Typhi—specific IgG antibodies against *S*. Typhi flagellar H antigen. The disease often does not show a specific clinical picture, and can be confused with other febrile illness such as malaria, dengue fever and *Staphylococcus aureus*. To overcome the problem of cross reactivity specific epitope of the flagellar H antigen was immobilised on the testing kit strip eliminating chances of cross reactivity and false positive results thereby increasing the specificity of the test. Since the immunodiagnostic kit, uses the flagellar H antigen from bacteria present in our country, the antibodies present in the serum of patients of our country will have maximum binding affinity, enhancing the sensitivity of our test kit. The immunodiagnostic kit on analysis gave a positive result with clinically diagnosed typhoid positive patient serum and negative results were obtained with the sera of clinically diagnosed malaria, abscess of *Staphylococcus aureus* and Visceral leishmaniasis (Kala-azar) patients.

## 1. Introduction

Typhoid fever is an enteric fever of humans caused by infection with *Salmonella enterica *serovar Typhi (*S*. Typhi). Although the isolation of *S*. Typhi on blood culture remains the gold standard for diagnosing typhoid fever, this may pose a major challenge in resource-limited settings where traditional laboratory methods of diagnosing typhoid are not available. India being an agriculturist economy, most of the population is concentrated in rural areas where most hospitals and primary health centres have no laboratory facilities; the diagnosis of typhoid fever is mostly based on clinical grounds, sometimes supported by the Widal test. Although, *S*. Typhi is a relatively invariant pathogen to antigenic variation, many of the surface antigens, therefore, may be conserved across the genera and induce antibodies that are cross reactive. Further, with an advancing age of an individual, he/she may accumulate cross reactive antibodies to *S*. Typhi. It means that it is just impossible to develop a specific diagnostic kit for typhoid using crude or semi-purified antigens. 

Essentially all serological tests for typhoid are based on the detection of antibodies to lipopolysaccharide (LPS) antigens (O9 and O12) [1–6]. As shown in the lateral flow assay for the detection of *S. *Typhi LPS specific antibodies, antibodies first start to develop at a time when the pathogen is already disappearing from the blood-stream [7]. The natural course and magnitude of the immune response seems to limit the sensitivity of serological testing for typhoid. Further, antigenicity of flagellar (H) proteins is higher than somatic (O) polysaccharide antigens [8]. Moreover, Brodie [9] has stated that agglutinins against flagella of *S*. Typhi are more frequent than O somatic (TO-9, 12) during an outbreak in Aberdeen in 1964.

Simple, reliable, point-of-care rapid diagnostic tests (RDTs) for typhoid fever have been a long-felt need of clinicians working in endemic areas [10]. They should be designed to yield a simple “positive/negative” result at thresholds pre-set by the manufacturers, similar to a pregnancy test. These results should normally be made available within 15 minutes, so that they can be used while the healthcare provider is dealing with suspected patients. Finally, such tests must be made available at low cost for use in resource-limited settings. Therefore, we planned to develop an indigenous immunodiagnostic kit using most dominant antigen epitope of flagellin protein of *S.* Typhi of North Indian origin and to test its specificity in different clinical specimens.

## 2. Materials and Methods

### 2.1. Isolation of Flagellar H: d Antigen Protein of *S*. Typhi

#### 2.1.1. Bacteria

Strain of *S*. Typhi (GNST 16/2008) obtained from Enteric Infection Unit of Department of Microbiology, Institute of Medical Sciences, Banaras Hindu University, India, was used for the preparation of flagellar H antigen.

#### 2.1.2. Bacterial Growth

Peptone water (1 gm peptone, NaCl 0.5 gm, distilled water 100 mL) was adjusted to pH 7.4 autoclaved at 121°C for 15 min was prepared. 200 mL Mueller-Hinton agar media was poured in the Roux bottle and allowed to solidify for 4 h. Fifty mL of the bacterial culture in peptone water was poured on the slant formed in the Roux bottle. The Roux bottle was shaken gently for 10 min at RT near the laminar flow and the excess peptone water was decanted. The Roux bottle containing the bacterial culture was incubated at 37°C for 48 h (without shaking). Thirty mL of normal saline (0.85% NaCl) was poured in the Roux bottle. It was shaken gently for 10 min near the laminar flow and the bacterial suspension was collected in the conical flask. Presence and purity of *S.* Typhi was checked by streaking on MacConkey Agar plate.

#### 2.1.3. Isolation of Flagellin Protein

The bacterial suspension was washed three times centrifuging at 12,000 rpm for 10 min by normal saline. The sediment from all the tubes was suspended in a total of 10 mL of normal saline. The suspension was then adjusted to pH 2.0 with 12 N HCl and constantly stirring for 30 min at RT. The bacterial cells, which were now devoid of flagella, were separated by centrifugation at 12,000 rpm for 30 min. The supernatant, which contained detached flagellin in monomeric form, was further centrifuged at 12,000 rpm for 1 h at 4°C. The pH of the supernatant was adjusted to 7.2 with 1 M NaOH. Ammonium sulphate was added slowly with vigorous stirring to achieve two-thirds saturation (2.67 M). The mixture was held overnight at 4°C and then centrifuged at 12,000 rpm for 15 min at 4°C. The precipitate, which contained flagellin, was dissolved in approximately one mL of dw and then transferred to dialysis tubing which had a molecular weight cutoff of 30,000 kDa (Sigma-Aldrich). Dialysis was carried out under running tap water initially for 2 h and then for 18 h at 4°C with constant stirring in 4 litres of distilled water containing 20 g of activated charcoal (Sisco Research Laboratories). The dialyzed flagellin preparations were then dissolved in 10 mM Tris and were estimated by Lowry's method [11].

#### 2.1.4. SDS-Polyacrylamide Gel Electrophoresis of Flagellin

The flagellar protein were analyzed by using SDS-polyacrylamide gel electrophoresis with slight modifications. Separating gel, 1.5 mm thick and 14 cm long, was prepared, consisting of 13% acrylamide, 0.325% bisacrylamide. Upon this, stacking gel of 3 cm length including wells, consisting of 5% acrylamide and 0.125% bisacrylamide, was performed. Final buffer composition in separating and stacking gels were 0.375 M Tris-HCl, pH 8.9, 0.1% SDS and 0.5 M urea and 0.125 M Tris-HCl, pH 6.7, 0.1% SDS and 0.5 M urea, respectively. These gels were polymerized chemically by the addition of 0.025% by volume of N,N,N′,N′-tetramethyl ethylene diamine (TEMED) and ammonium persulfate (250 *μ*g/mL). The electrophoresis buffer (pH 8.3) contained 0.025 M Tris, 0.192 M glycine, and 0.1% SDS and 0.5 M urea. The samples were mixed with 1x sample buffer to have finally 0.06 M Tris, pH 6.7, 2% SDS, 10% glycerol, 0.001 bromophenol blue, and 0.1% by volume beta-mercaptoethanol just before loading. The proteins were completely dissociated by heating the samples in boiling water bath for 90 s. The electrophoresis was performed at 150 V and was stopped after the dye eluted out of the gel. Gels were placed in 20% TCA for 30 min to fix the proteins. Gels were stained with Coomassie blue R-250 (0.3% w/v) in 50% methanol and 7.5% acetic acid overnight and destained with solution having 30% methanol, 7.5% acetic acid. After destaining, the gels were stored in 10% acetic acid.

### 2.2. Screening for Antigenicity of Isolated Flagellin Protein

The isolated protein (3 *μ*g/5*μ*L) w/v was immobilized to polyvinylidene fluoride sticks and blocked with 3% gelatin for 2 h at 37°C. The sticks were washed 3 times with PBS/T. The antigen coated polyvinylidene fluoride sticks were incubated in 500 *μ*L volume of diluted (1 : 100 in PBS/T) typhoid positive (culture confirmed) and negative sera for 1 h at 37°C in small plastic vials. Sticks were washed 3 times PBS/T (for washing added PBS/T, shook gently and discarded after 3 min). Each stick was incubated in 500 *μ*L of optimally diluted (1 : 10,000) anti-human IgG horse radish peroxidase conjugate for 1 hour at 37°C. The sticks were washed with PBS/T five times (as described earlier). After washing, the strips were incubated with diaminobenzidine substrate for 5–10 min at 37°C. The reaction was stopped using distilled water. The reaction was read by colour change.

### 2.3. Epitope Isolation of Flagellin Protein of *S*. Typhi

#### 2.3.1. Enzyme Hydrolysis of Flagellin Protein of *S*. Typhi

One milligram amount of flagellin protein was suspended in 1.25 mL of 10^−3^ M tris(hydroxymethyl)aminomethane (tris)-hydrochloride buffer to give a final pH of 8.1 when hydrolysis by trypsin and chymotrypsin was followed. The reaction mixture was incubated at 37°C in a shaking water bath for 60 min for digestion with trypsin was and for 20 min for digestion with chymotrypsin (trypsin and chymotrypsin were in a 1 : 50 (w/w) ratio to protein). The enzyme trypsin was obtained from New England Bioloabs, (MA, USA) and the enzyme Chymotrypsin from Sisco Research laboratories (India). Trypsin treated with l-(1-tosylamido-2-phenyl) ethyl chloromethyl ketone (TPCK) was used.

#### 2.3.2. SDS-PAGE Employing Protease-Hydrolysed Flagellin Protein

 Electrophoresis was carried out on sodium dodecyl sulphate (SDS)-acrylamide gels essentially described by Weber and Osborn [12]. Gels were prepared with 10% acrylamide and 0.25% bisacrylamide. Samples of enzymatically hydrolyzed flagellin (usually 20 to 50 *μ*L) were dissociated in 10^−3^ M sodium phosphate buffer (pH 7.2) containing 2% sodium dodecyl sulphate and 2% beta-mercaptoethanol by heating in a boiling water bath for 2-3 min. The electrophoresis was performed at 150 V and was stopped after the dye eluted out of the gel. Gels were placed in 20% trichloroacetic acid for 30 min to fix the proteins. Gels were stained with Coomassie blue R-250 (0.3% w/v) in 50% methanol and 7.5% acetic acid for overnight and destained with solution having 30% methanol, 7.5% acetic acid. After destaining, the gels were stored in 10% acetic acid.

#### 2.3.3. Western Blotting Employing Hydrolysed Flagellin Protein

Enzymatically hydrolyzed flagellin protein were separated on 10% SDS-PAGE and were electrophoretically transferred to Polyvinylidene fluoride membranes (Pierce Biotechnology, Rockford, IL, USA or Bio-Rad Laboratories, CA, USA) by using Nova Blot semidry system (Multiphor II & EPS 600, Amersham Pharmacia Biotech, NJ, USA) as per manufacturer's instructions. The membranes were blocked with 5% bovine serum albumin in 10 mM Tris-HCl, 150 mM NaCl, pH 8.0, (Tris -buffer saline) containing 0.05% Tween-20 for 1 h at RT. Blots were then incubated for 1 h at 4°C with serum samples of patient cases suffering from typhoid fever, visceral leishmaniasis, *Staphylococcus aureus* abscess, and malaria (1 : 100 dilutions in 2% BSA in 1 × TBS-T) acting as primary antibodies. Following washing, the blots were incubated for 1 h with horse radish peroxidase-labeled secondary antibody (1 : 10,000 dilutions in 2% BSA in 1 × TBS-T), that is, anti-human IgG-HRP conjugate. Following washing, the enzyme activity on polyvinylidene fluoride membrane was revealed by developing the colour with freshly prepared 3,3-diaminobenzidine solution (0.05 mg dissolved in 1 mL of 50 mM citrate buffer, pH 5.6, containing 0.03% H_2_O_2_). The reaction was stopped using distilled water.

### 2.4. Preparation and Testing the Kit

The test kit prepared contained buffer well (B), sample well (A), test well (T), and the control well (C). To the test well “H” antigen flagellin protein epitope was coated, and control well had human IgG immobilised. The nitrocellulose membrane (Millipore HF Plus 135, USA) was then saturated with BSA (30%), after coating and dried by incubation for 2 h at 40°C. Serum (~5 *μ*L) was applied in the sample well followed by addition of five drops of buffer (0.01 M Tris buffer with 0.1% sodium azide) in the buffer well and buffer was allowed to run off the membrane. Thereafter, colloidal Gold-conjugated anti-human IgG (~5 *μ*L) was applied in the sample well followed by addition of 5 drops of buffer in the buffer well. After buffer had run off the nitrocellulose membrane, antibody binding was detected by coloured (burgundy colour) line on the test kit. 

## 3. Result

### 3.1. SDS-Polyacrylamide Gel Electrophoresis of Flagellin Protein

**Figure 1:**
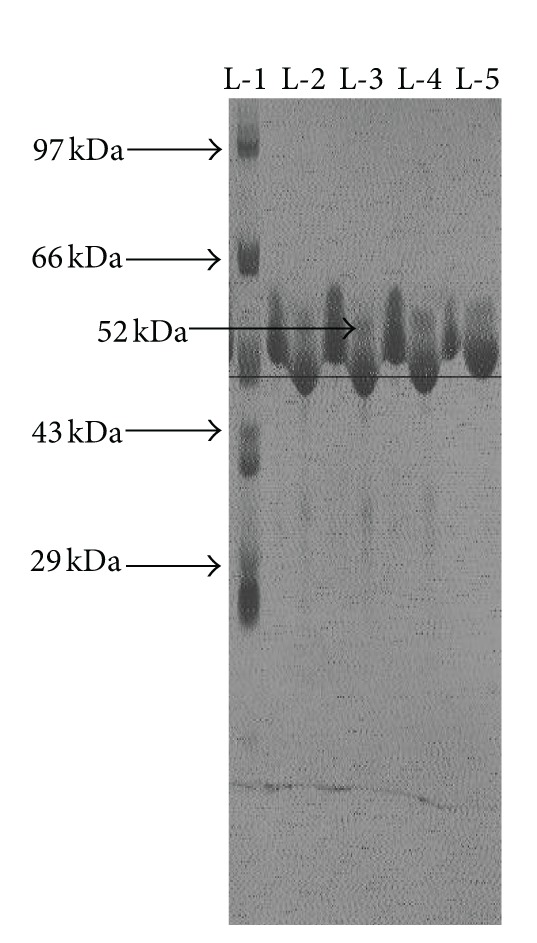
Lane 1: standard molecular weight marker, lanes 2–5: flagellar antigen (approx. 52 kDa).

Flagellin yielded a single stained band in polyacrylamide gel electrophoresis, indicating the probable purity of the protein isolated. The flagellar protein (flagellin) mass was determined to be ~52 kDa as is evident from the Figure 1.

### 3.2. Detecting Antigenicity of Isolated Flagellin Protein by Stick ELISA

**Figure 2:**
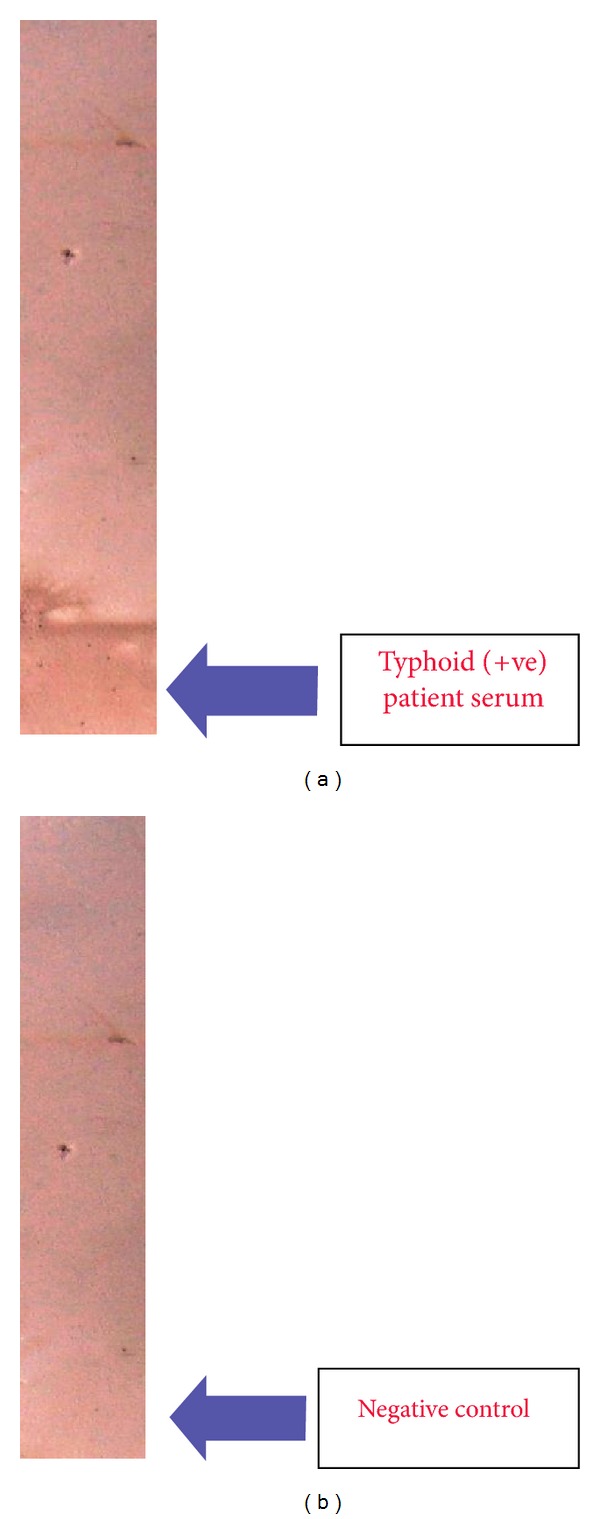
(a) Polyvinylidene fluoride sticks incubated with typhoid-positive serum, (b) polyvinylidene fluoride sticks incubated with typhoid-negative serum.

The result of the sick ELISA demonstrates the presence antibodies to the flagellar (H) antigen protein in the serum from patient with typhoid infection (clinically positive for *S.* Typhi) Figure 2(a) and no antibodies were detected in serum from patient (clinically negative for *S.* Typhi) Figure 2(b). Thus, this result strongly suggests that the flagellar (H) antigen protein of *S*. Typhi is a strong immunogen in vivo and would be highly useful for the detection of typhoid fever.

### 3.3. Detection of Antigenic Epitope of Flagellin Protein

**Figure 3:**
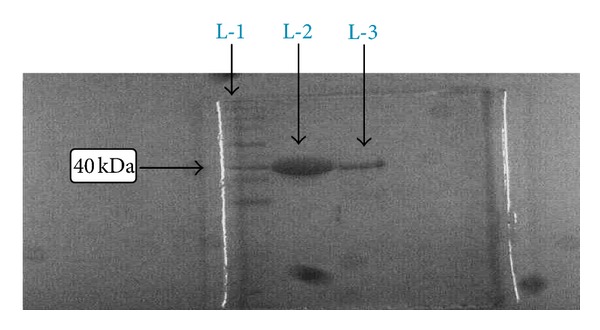
Enzymatic digestion of *S*. Typhi flagellin protein. L-1: Standard protein molecular weight marker. L-2: Trypsin digestion product. L-3: Chymotrypsin digestion product.

Figure 3 shows the proteolysis of isolated flagellin protein (molecular weight ~52 kDa). On standardisation, by 60 min of trypsin treatment (Figure 3, lane L-2), the original flagellin band was no longer detected on the gels and a band with an approximate molecular weight of ~40 kDa (antigenic epitope) was the major one seen. On the other hand, the other serine protease, chymotrypsin, on standardisation, too had a similar effect; when 20 min of the chymotrypsin treatment (Figure 3, lane L-3) was carried out on isolated flagellin protein. 

Lower molecular weight products of hydrolysis were formed, but they escaped detection by the Coomassie Brilliant Blue staining method.

### 3.4. Western Blotting for the Antigenic Epitope

The specificity and avidity of antibodies present in our sera of interest including the controls, for flagellar (H) antigen epitopic protein was analysed by the technique of a dot-immunobinding assay. 

**Figure 4:**
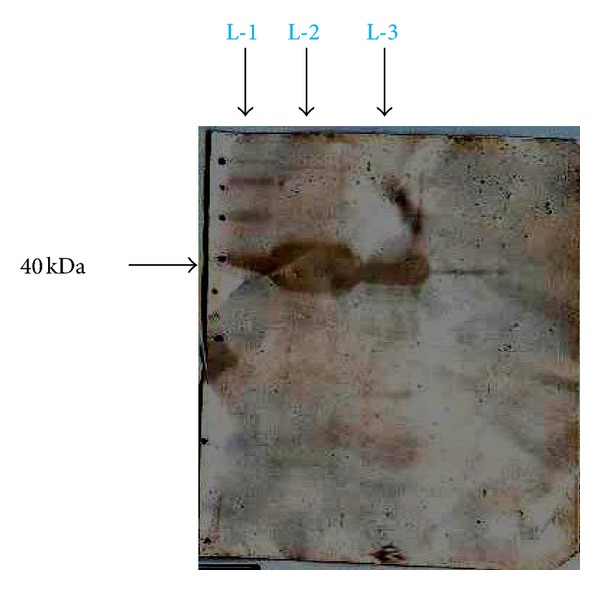
Typhoid positive patient serum, L-1: standard protein molecular weight marker, L-2: trypsin digestion product, L-3: chymotrypsin digestion product.

**Figure 5:**
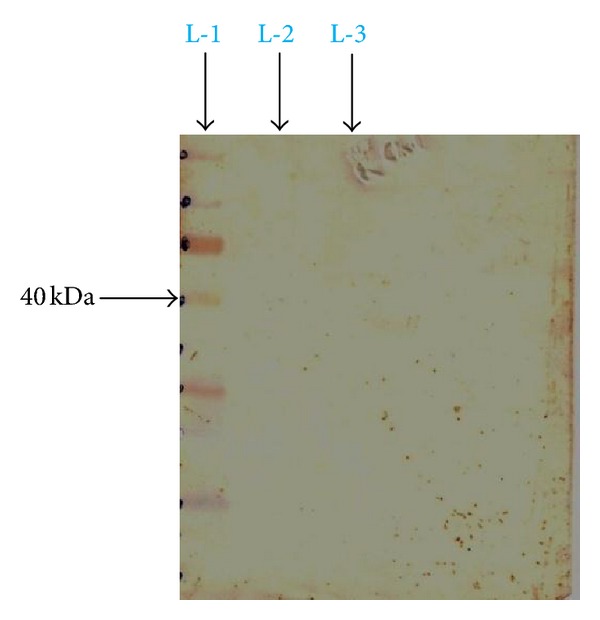
Malaria-positive patient serum, L-1: standard protein molecular weight marker, L-2: trypsin digestion product, L-3: chymotrypsin digestion product.

**Figure 6:**
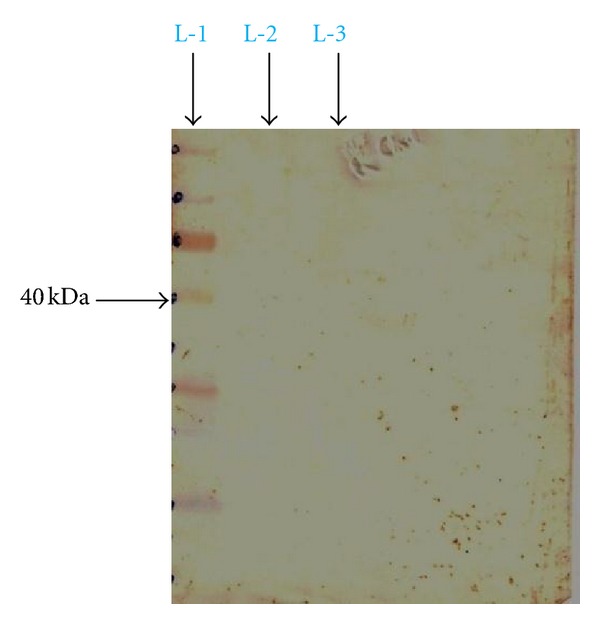
Patient suffering from *Staphylococcus aureus* abscess serum, L-1: standard protein molecular weight marker, L-2: trypsin digestion product, L-3: chymotrypsin digestion product.

**Figure 7:**
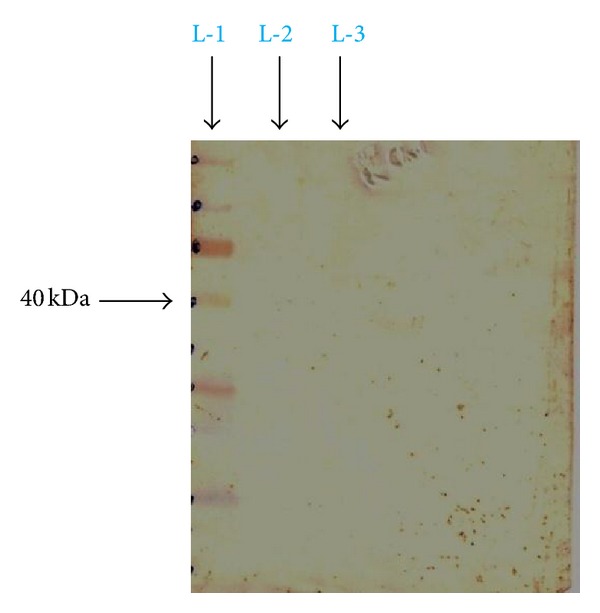
Kala-azar-positive patient serum, L-1: standard protein molecular weight marker, L-2: trypsin digestion product, L-3: chymotrypsin digestion product.

On analysis of the result obtained from the immunodotblot (Figure 4), the antibodies present in typhoid-positive patient serum were hardly able to recognize the lower molecular weight peptides, and this could be visualised by the nearly faint intensity of colour developed in the lower of half of the immunodotblot. On the other hand, the upper half of the immunodotblot shows markedly a very strong recognition of antibodies present in typhoid patient serum to the ~40 kDa flagellar (H) antigen peptide band. But when the of sera of control cases (malarial, abscess due to *Staphylococcus aureus*, and visceral leishmaniasis) were used one could clearly figure out from the immunodotblots (Figures 5, 6, and 7) that the sera antibodies from all the three control patients did not recognize any of the major or the minor faint flagellar (H) antigenic bands on the immunodotblot. 

### 3.5. Result of Test Kit

In an effort to develop a rapid, reliable, specific, and sensitive test for the diagnosis of typhoid fever, we attained a test kit with conspicuous results. 

**Figure 8:**
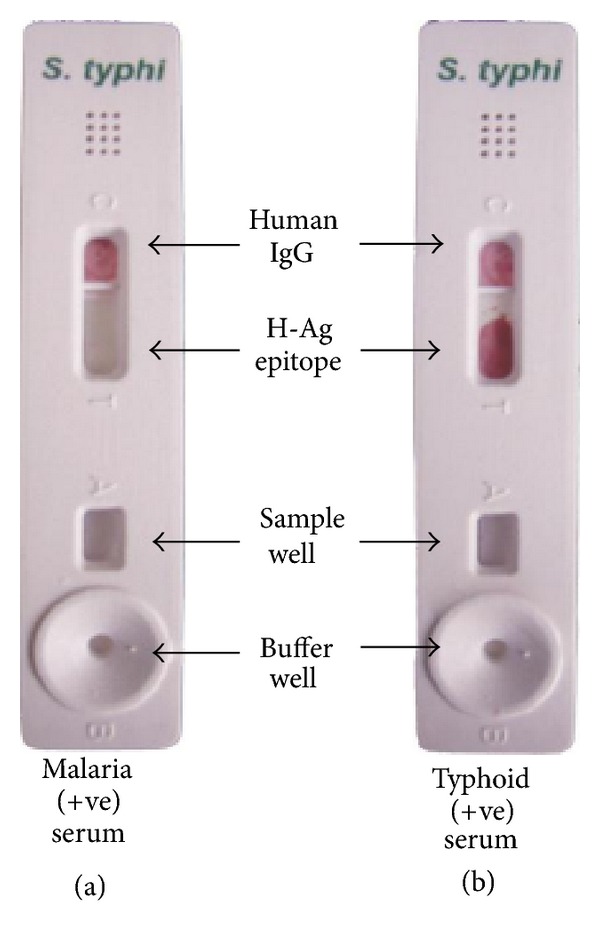


**Figure 9:**
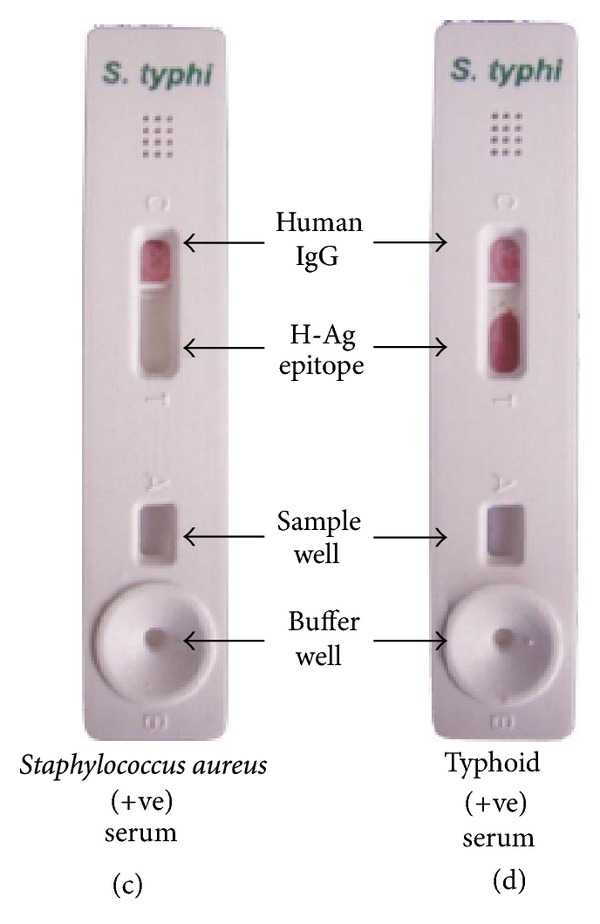


**Figure 10:**
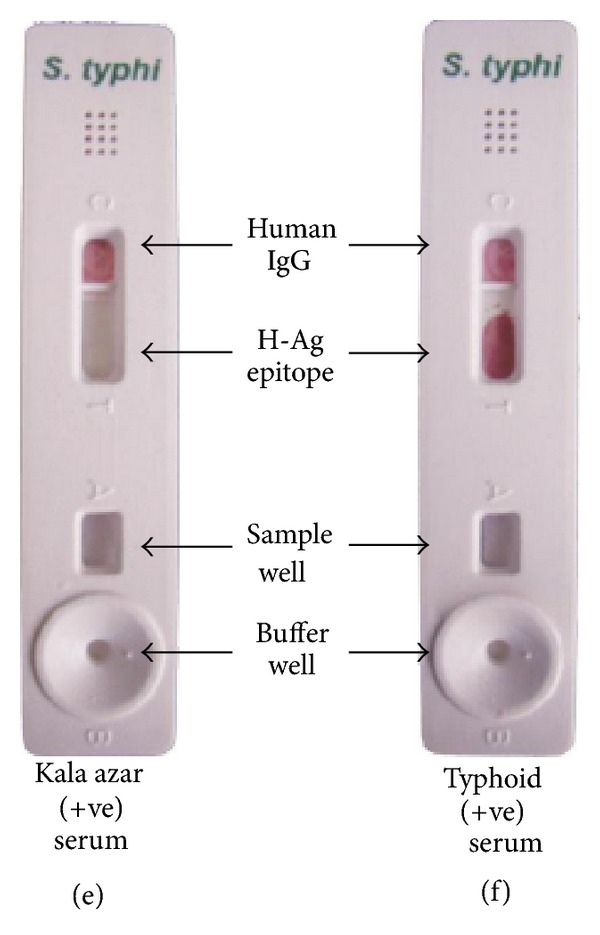


The test kit gave a positive result with the serum of typhoid fever positive patient (clinically confirmed) acting as “true positives”; who was admitted during the second week of illness, in the Sir Sunderlal Hospital, BHU, Varanasi where the study has been carried out and patient had no antibiotic therapy administered. The test kit gave negative results when the sera of control cases (malarial, abscess due to *Staphylococcus aureus*, and visceral leishmaniasis) were used “acting as controls and true negatives” as in (Figures 8, 9, and 10). The test is invalid if the control band is not visible within 15 min as in Figure 11.

## 4. Discussion

The prevalence of *S.* Typhi infection differs in different parts of the World. In the developing countries like India, *S.* Typhi infection is more frequent among general population. In this study explanation has been done for the possible use of flagellin protein epitope to use for diagnostic kit development. For this purpose a ~52 kDa flagellin protein from indigenous strain of *S*. Typhi was isolated and was found to be antigenic. Our finding is well supported by Anuntagool et al. [13] who also could purify ~52 kDa protein from *S*. Typhi flagella. Sukosol et al. [14] could raise a monoclonal antibody also to detect the same *S*. Typhi specific ~52 kDa antigen of *S*. Typhi. Further Korbsrisate et al. [15] has also reported utility role of a *S*. Typhi flagellar protein of ~52 kDa in serodiagnosis of typhoid fever. On hydrolysis by serine proteases trypsin and chymotrypsin, a band of ~40 kDa protein (antigenic) epitope could be observed, while other bands were invisibly small on SDS-PAGE analysis. There is a report showing that terminal regions of flagellin protein are very sensitive to proteolysis, resulting into small oligopeptides by tryptic digestion [16] and yielding a fragment of ~40 kDa. Here it is interesting to mention that flagellins present in the members of family Enterobacteriaceae and Campylobacter species produce the similar ~40 Kda protein fragment on cleavage by chymotrypsin. On Western blotting of ~40 kDa band by the use of serum from typhoid patient showed very strong colour development, indicating strong antigenicity of the fragment. When the specificity of the ~40 kDa band was checked by using sera from patients suffering from acute typhoid fever, malaria, kala-azar, and abscess due to *Staphylococcus aureus*, only serum from acute typhoid fever reacted well while others did not give the desired colour development. On getting these encouraging results, a test kit was developed by coating the ~40 kDa epitope on a nitrocellulose membrane strip (Millipore HF Plus 135) and detected specific antigen-antibody reaction by chromogenic assay (colloidal Gold conjugated human immunoglobulin). The test was carried out on the serum as mentioned earlier and interestingly the positive could be observed only with the serum from typhoid fever patient while others (malaria, kala-azar, and abscess due to *Staphylococcus aureus*) did not produce coloured band. It is important to mention here that the present kit has been produced by the flagellin epitope extracted from the local *S*. Typhi strain. The present kit is based on chromogenic method requiring no special equipments and easy to perform in the rural field conditions. The methodology developed in the present study can be used to prepare very sensitive and specific indigenous kit with specific antigen of somatic, flagellar, and Vi coated on the same kit and enabling us to use it in endemic areas like India. However, this kit needs to be evaluated in large number of sera collected from cases and controls to see its sensitivity and specificity.

## Figures and Tables

**Figure 11:**